# Spatiotemporal Patterns of Crested Ibis (*Nipponia nippon*) Movement

**DOI:** 10.3390/ani15172555

**Published:** 2025-08-30

**Authors:** Zhengyang Qiu, Ke He, Shidi Qin, Wei Li, Chao Wang, Dongping Liu

**Affiliations:** 1 School of Computer Science and Technology, Beijing Jiaotong University, Beijing 100044, China; zhengyangqiu@bjtu.edu.cn; 2 College of Animal Science and Technology, College of Veterinary Medicine, Zhejiang Agriculture and Forestry University, Hangzhou 311300, China; 3 Key Laboratory of Biodiversity Conservation of National Forestry and Grassland Administration, Ecology and Nature Conservation Institute, Chinese Academy of Forestry, Beijing 100091, China; 4 Shaanxi Hanzhong Crested Ibis National Nature Reserve, Hanzhong 723300, China

**Keywords:** *Nipponia nippon*, site fidelity, movement ecology, habitat partitioning, long-term tracking, conservation planning

## Abstract

Understanding long-term movement ecology is critical for conserving endangered species; however, comprehensive spatiotemporal analyses remain limited. In this study, we leveraged a decade-long GPS tracking dataset (2014–2024) of 31 endangered Crested Ibis (*Nipponia nippon*) individuals to elucidate their spatiotemporal behavioral patterns. The study focused on three key aspects: (1) fidelity to nesting, foraging, and roosting sites; (2) movement patterns and their ecological drivers; and (3) foraging habitat preferences across regions and activity periods. The results revealed exceptional fidelity to nesting, foraging (mean value = 0.253), and roosting sites (mean value = 0.261), underscoring the species’ pronounced spatial memory. Temporal factors emerged as the primary drivers of movement patterns, demonstrated by a significant annual reduction in home range size (*p* < 0.01) and a decline in daily flight distance in 2019 (β = −1890 ± 772 m, *p* < 0.05) and 2022 (*p* = 0.052). Behavioral factors also significantly influenced daily flight distance, with notable variations across different activity periods. Foraging habitat selection exhibited considerable spatial heterogeneity (14.2% constrained variance, *p* < 0.01). Cultivated lands, particularly paddy fields (Yangxian population) and drylands (Tongchuan population), served as core foraging zones. In contrast, spatiotemporal variables such as age had limited effects (<5% variance). This study provides the first empirical evidence of long-term site fidelity and habitat partitioning in the Crested Ibis, emphasizing the importance of landscape-level conservation planning. To this end, we propose two targeted strategies: establishing habitat corridors to enhance connectivity and safeguarding stable foraging areas within agricultural landscapes. These findings contribute to movement ecology theory while offering actionable frameworks for endangered species management.

## Simple Summary

We utilized a decade-long dataset to analyze the spatiotemporal dynamics of the movement ecology of the endangered Crested Ibis (*Nipponia nippon*), emphasizing habitat fidelity and landscape utilization. Our findings reveal exceptional inter-annual site fidelity in foraging, roosting, and nesting behaviors and identify significant spatiotemporal drivers of habitat selection. Additionally, we underscore the ecological significance of cultivated lands, particularly paddy fields and drylands, as core foraging zones.

## 1. Introduction

The Crested Ibis (*Nipponia nippon*) is listed as Endangered (EN) on the International Union for Conservation of Nature (IUCN) Red List (2024) [1], primarily due to historical habitat loss caused by deforestation, wetland degradation, and agrochemical use in rice paddies, which reduced prey availability [2]. The population has rebounded from just seven individuals in Yang County, Shaanxi Province, in 1981 [3] to approximately 11,000 individuals worldwide by the end of 2024. This remarkable recovery is largely attributed to coordinated conservation efforts, including habitat monitoring in locally protected areas and captive breeding programs. Stable wild populations have now been established in multiple regions worldwide, including Yang County, Ningshan, and Tongchuan in Shaanxi Province; Dongzhai in Henan Province; Deqing in Zhejiang Province; as well as in South Korea [4] and Japan [5]. The long-term sustainability of these populations depends largely on their ability to effectively utilize local habitats. The Crested Ibis is a non-migratory, colonial-nesting species. It remains highly social throughout the year, feeding and roosting in groups. Its diet consists of fish, amphibians, and insects.

Historically, habitat use by the Crested Ibis has been determined through field surveys and navigational positioning technologies [6]. These field investigations include baseline surveys and continuous monitoring, involving routine patrols of core activity areas, assessments of breeding activity, and surveys of roosting sites to track population dynamics [6]. However, the species’ rapid population growth and substantial habitat range expansion have posed considerable challenges to traditional manual habitat surveys (e.g., roosting and foraging areas). These challenges include high labor demands, time constraints, and increased operational costs. Moreover, conventional methods lack the capacity to deliver real-time movement trajectories or to identify potentially suitable habitats, thereby hindering timely habitat protection efforts. The adoption of wearable GPS tracking devices has emerged as an efficient solution for collecting real-time movement data in navigation systems [7,8].

Animal tagging, positioning, and tracking have become critical methods for investigating spatial movement ecology. Sensor-based tracking technologies are widely used in wildlife research, particularly in studies of habitat selection and use [8], behavioral and diurnal activity patterns [9], and migration (e.g., Whimbrel [10,11]). Previous studies have demonstrated that this method is applicable to the Crested Ibis [12,13,14]. Jiang et al. (2022) [12] successfully identified habitats by analyzing the spatial characteristics of individual movement paths, enabling precise differentiation between roosting and foraging sites across multiple locations. Zhou et al. (2023) [13] revealed distinct habitat distribution patterns using data from seven individuals, while additional investigations assessed the influence of environmental factors such as vegetation, elevation, and proximity to rivers on habitat selection. In South Korea, researchers have employed tracking data to analyze breeding behavior patterns [4]. However, most of these analyses have been limited to short-term observations and lack a comprehensive examination of temporal dynamics.

In our previous studies, we developed a visual analysis system to track the movement trajectories of individual Crested Ibis across multiple spatiotemporal dimensions [14]. Additionally, we established a robust long-term monitoring program for wild Crested Ibis populations [14]. In this study, we leveraged a decade-long dataset (2014–2024), encompassing multiple populations, to address three key research objectives: (1) to identify nesting, primary foraging, and roosting sites, and analyze site fidelity; (2) to characterize movement patterns through assessments of home range and daily flight distance; and (3) to determine foraging habitat types and assess associated ecological drivers. To our knowledge, this is the first study to systematically analyze the spatial ecological characteristics of the Crested Ibis based on long-term population data. The findings are expected to provide a theoretical foundation for advancing conservation strategies and guiding future management efforts for this species.

## 2. Materials and Methods

### 2.1. Study Area and Tag Deployment

**Figure 1:**
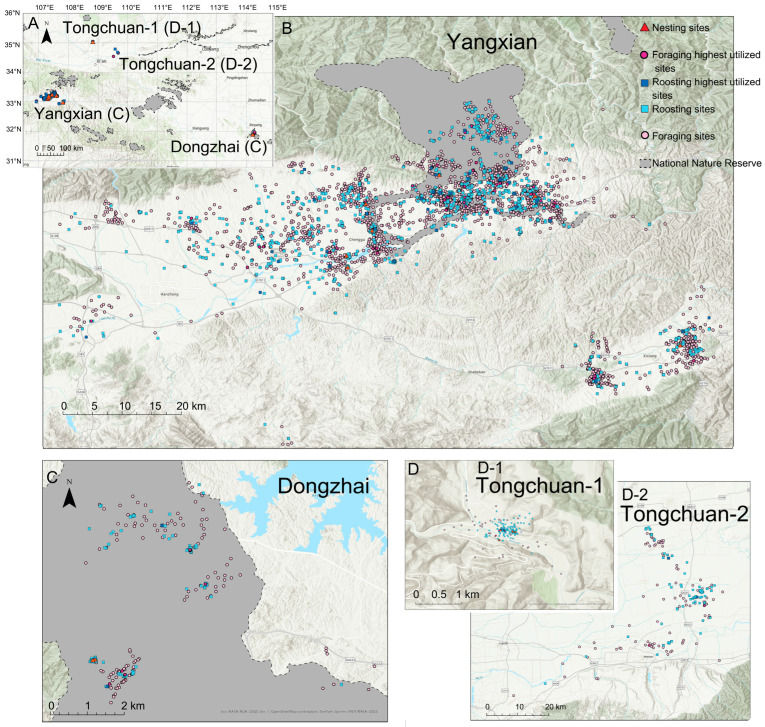
(**A**) Location of three study areas and associated Crested Ibis foraging, roosting, and nesting sites. More detailed views of each study area are provided in (**B**) Dongzhai, (**C**) Yanxian, and (**D**) Tongchuan. Since the two regions of Tongchuan were somewhat far apart, -1 and -2 were used to represent them. Highest utilized sites represent the most frequently used roosting and foraging locations for each individual bird annually.

The study was conducted from 2014 to 2024 in three Crested Ibis populations in China: Yangxian (YX) and Tongchuan (TC) in Shaanxi Province, and Dongzhai (DZ) in Henan Province (Figure 1). Individuals monitored for more than 100 days were selected for analysis, resulting in a total of 31 monitored individuals (Table S1). Individuals were categorized into two age groups: juvenile (<2 calendar years) and adult (≥2 calendar years).

GPS-GSM transmitters (HQBN2525 and HQBN3527; Hunan Global Messenger Technology Co., Ltd., Changsha, China) were attached to individuals following the methodology described in our previous study [6]. The combined weight of the transmitter and harness accounted for 1.6–1.9% of each individual’s body mass. All transmitters were solar-powered and programmed to record positional data at one-hour intervals.

### 2.2. Identification of Nocturnal Roosting, Foraging, and Nesting Sites

First, only locations considered accurate within 5–100 m were used in the analysis, as recommended by the manufacturer. Second, nesting sites, nocturnal roosting sites, and foraging sites were analyzed using filtered data where the recorded flight speed was less than 5 km/h, as our tracking device provided real-time flight speed values and records with speeds below this threshold were treated as stationary. Some time periods represent transitions between diurnal foraging behavior and nocturnal roosting behavior. To reduce potential error, locations recorded during these transitional periods were excluded from the analysis. Three biologically defined activity periods were used: the breeding period (February–June), wandering period (July–October), and wintering period (November–January), along with roosting and foraging time periods. This classification is based on the Crested Ibis’ ecological patterns: they typically select breeding sites near forested mountainous areas with abundant arboreal resources and minimal human disturbance. Post-breeding, individuals gradually disperse from breeding grounds to plains for overwintering. The wandering period thus represents a dispersal phase with distinct foraging and roosting site selection patterns compared to breeding and wintering periods.

Geographic coordinates were analyzed using a distance matrix, and hierarchical clustering was performed based on predefined thresholds using the “geosphere” package in R (version 4.4.1) [15]. Foraging sites were defined as clusters of locations during foraging periods where all points were within 500 m of each other, while nocturnal roosting sites were defined as clusters within 50 m during roosting periods [14]. Nesting site identification was assessed during the breeding season and required meeting two criteria: (1) the individual was classified as an adult during the period, and (2) the individual had at least 20 daytime records within a single site (distance threshold: 50 m), as well as at least 20 nighttime records indicating nesting presence. This dual requirement ensured the incubation and feeding behavior during nesting. Locations meeting both criteria were designated as nesting sites. Field investigations were conducted after the breeding season to validate the accuracy of the identified nesting locations. Once clusters for the three site types (nocturnal roosting, foraging, and nesting) were identified, the average latitude and longitude of the points within each cluster were calculated and used as representative site locations. We then analyzed foraging and nocturnal roosting sites according to the breeding, wandering, and wintering periods each year. Each individual × year combination from long-term monitored individuals was treated as an independent observational event for computing foraging and roosting locations. Statistical comparisons of multiple parameters, including the number of foraging and nocturnal roosting sites, as well as the fidelity of each site type, across different activity periods were performed using Mann–Whitney U tests with Bonferroni correction for multiple comparisons.

Fidelity for each individual was analyzed as follows. First, nest-site fidelity was quantified using individuals with multi-year nesting site records. Each confirmed nesting event was treated as a discrete observational unit. Nest-site consistency was determined by comparing nesting locations across years, with sites located within a 50 m spatial threshold considered the same nesting site. The nest-site fidelity index was calculated as: Nest-site fidelity = Number of times at the same nest site/Total number of recorded nest sites. Next, to assess fidelity to nocturnal roosting and foraging sites, we calculated the utilization frequency of each site as the number of location records at the site divided by the total number of location records within each independent observational event (defined as an individual × year combination). The site with the highest frequency was selected as the fidelity value for that event.

### 2.3. Movement Pattern: Home Range and Daily Flight Distance

Crested Ibis movement patterns were assessed using home range estimates and daily flight distances. Home ranges were determined using the 50%, 90%, and 95% utilization distributions (UDs), estimated via kernel density estimation (KDE) implemented in the “adehabitatHR” package in R. The kernelUD function was applied with a reference bandwidth (“href”) to compute UDs, and 95% UD contours were extracted using the getverticeshr function. The resulting spatial polygons were converted into “sf” objects for geometric validation, and their areas were calculated and reported in square kilometers (km^2^). Monthly home ranges were calculated for each individual, and only months in which the number of recorded days exceeded half the total days in the month were included in the analysis.

Daily flight distances were calculated from chronologically ordered daily GPS locations. Individuals with insufficient tracking data (<15 days/month) were excluded to ensure reliable monthly average flight distance estimates. The average daily flight distance was computed for each month; months with more than half of the days unrecorded were excluded from further analysis.

A linear mixed model (LMM) was used to assess the effects of Site (YX, TC, and DZ), Age group (juvenile and adult), Year, and Activity period (breeding, wandering, and wintering) on home range size (KDE area_95) and daily flight distance. Years with insufficient data points (<20 records), including 2014, 2023, and 2024, were excluded from the analysis. Outliers were removed based on the 3σ principle. KDE area_95 was modeled as the response variable, with Site, Age group, and Activity period as fixed effects. A similar model was used with daily flight distance as the response variable to evaluate the relative contribution of each factor to variation in monthly movement patterns.

### 2.4. Foraging Habitat Selection with Redundancy Analysis

The habitat type dataset was obtained from the Data Center for Resources and Environmental Sciences at the Chinese Academy of Sciences (RESDC; http://www.resdc.cn). To analyze foraging habitat selection, we calculated two parameters: visit frequency (proportion_day = visits to the target site/total behavioral records) and visit persistence (proportion_count = days with ≥1 visit/total observation days). Correlation analysis revealed a strong relationship between the two metrics (r = 0.987–0.996); therefore, proportion_count was selected for further analysis. Statistical comparisons of habitat selection across the three activity periods were conducted using the non-parametric Kruskal–Wallis test. When significant differences were found (*p* < 0.05), pairwise comparisons were performed using Wilcoxon rank-sum tests.

To assess the influence of environmental and temporal variables, including Site, Year, Age group, and Activity period, on foraging site selection, redundancy analysis (RDA) was employed. All statistical analyses were conducted using R software. RDA was performed using the rda() function from the “vegan” package, and the relative contributions of explanatory variables were evaluated using hierarchical partitioning via the “rdacca.hp” package.

## 3. Results

### 3.1. Identification and Evaluation of Fidelity to Nesting, Foraging, and Nocturnal Roosting Sites Based on Tracking Data

Our analysis identified 14 distinct nesting sites, 2201 foraging sites (range: 4–294 per individual), and 2532 nocturnal roosting sites (range: 9–367 per individual), based on GPS tracking data from 31 individuals collected throughout the study period (Figure 1, Tables S1 and S2).

Nesting Sites. The dataset included 32 breeding periods from 14 individuals; 17 individuals were excluded due to a lack of breeding data. All 14 nest sites were verified through field surveys (Table S3). Nest-site fidelity was recorded for three individuals across multiple years. Two individuals (CAFL003 and 4B04A0) exhibited 100% site fidelity. Notably, individual CAFL003 returned to the same forest area (inter-nest distances < 200 m) for six consecutive breeding seasons (2015–2020), indicating remarkable site fidelity. In contrast, CAFL031 nested in two locations nearly 2 km apart during the 2020 and 2021 breeding seasons.

**Figure 2:**
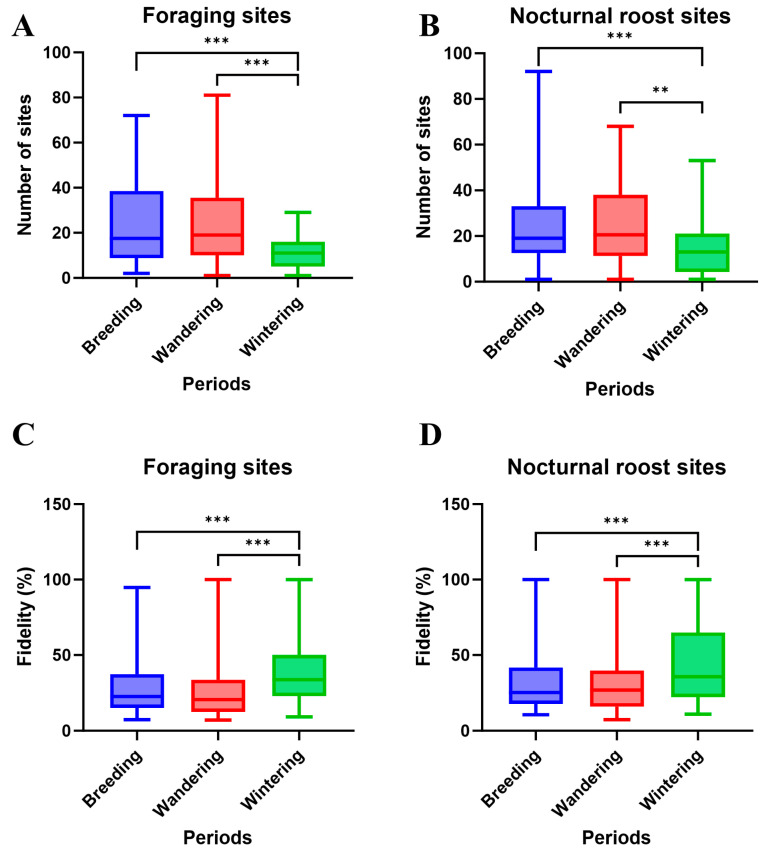
The site number of (**A**) foraging and (**B**) nocturnal roost sites; and site fidelity of (**C**) foraging and (**D**) nocturnal roost sites. ** *p* < 0.01, *** *p* < 0.001.

The number of foraging and roosting sites varied across the three activity periods, wintering, wandering, and breeding (Table 1, Figure 2A,B). Wintering periods had fewer foraging sites (mean = 11.45) and roosting sites (mean = 13.83) compared to both the breeding period (foraging: 23.54; roosting: 23.96; Wilcoxon test, *p* < 0.001) and the wandering period (foraging: 24.46; roosting: 26.37; Wilcoxon test, *p* < 0.001). Although the breeding period showed fewer sites than the wandering period, the difference was not statistically significant (Wilcoxon test, *p* > 0.05). The results also revealed substantial fidelity in both foraging and roosting behaviors (Table 1). For the most frequently used foraging sites per individual per year, the median fidelity was 0.253 (*n* = 73 individual-year events), with 9 events (12.33%) exceeding 0.500. For nocturnal roosting sites, the median fidelity was 0.261 (*n* = 72), with 5 events (6.94%) exceeding 0.500. Site fidelity remained consistently high across breeding, wandering, and wintering periods (Table 1). However, significant variation in fidelity strength was observed across periods (Figure 2C,D). Both foraging and roosting site fidelity were significantly higher during the wintering period than during the wandering period (*p* < 0.01) and breeding period (*p* < 0.001). No significant differences were observed between the breeding and wandering periods. These findings indicate strong spatial preferences and high site fidelity in Crested Ibis resource use, particularly during wintering periods.

Analysis of interannual variation in site usage revealed notable temporal consistency in site fidelity. Among the most frequently utilized foraging and nocturnal roosting sites, 26 of 61 foraging events and 24 of 61 roosting events exhibited stable site usage across years. These events occurred in 9 of the 19 individuals for whom multi-year tracking data were available. In certain cases, such as individual CAFL003, exclusive site use was observed; this individual consistently used the same dominant location for foraging in all six recorded years and for nocturnal roosting in five of six years.

When combined with the boundaries of the national nature reserve, the spatial analysis revealed distinct patterns among populations. Most DZ individuals remained within the protected area (Figure 1B), whereas YX individuals showed partial dispersal beyond the reserve boundary (Figure 1C). In contrast, all TC individuals were distributed exclusively outside the reserve (Figure 1D).

### 3.2. Movement Pattern and Related Ecological Factors

#### 3.2.1. Movement Pattern of Daily Flight Distance and Monthly Home Range Size

The daily and seasonal movement patterns of Crested Ibis individuals were quantified using two key metrics: monthly average daily movement distance and monthly home range size (area_95). The results revealed substantial individual variation in both movement parameters (Table 2). The monthly home range sizes (area_95) across different activity periods ranged from 7.76 to 15.45 km^2^, while median daily movement distances varied between 2952 and 3081 m. Statistical analyses indicated no significant differences in either parameter across the three activity periods (all *p* > 0.05). Given the lack of significant influence from activity periods, we applied linear mixed models (LMM) to explore the ecological drivers of movement behavior.

#### 3.2.2. Ecological Factors Influence Movement Patterns

**Figure 3:**
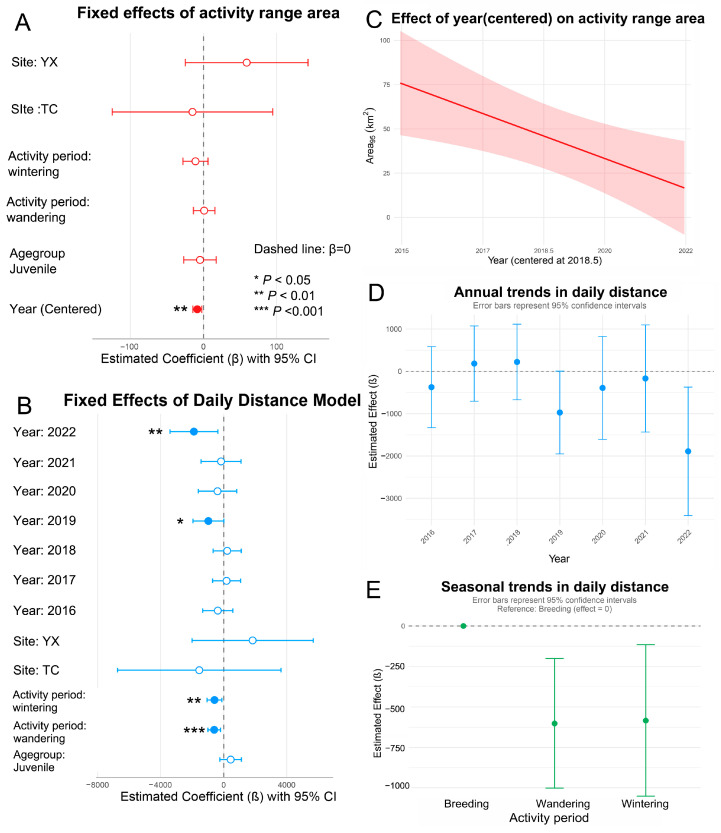
Results from the mixed-effects models analyzing movement patterns. (**A**) Fixed effects estimates for activity range (95% area); (**B**) Fixed effects estimates for daily movement distance. In (**A**,**B**), dashed lines indicate null effects (β = 0) along with 95% confidence intervals; (**C**) Annual trends in activity range (95% area); (**D**) Seasonal variation; and (**E**) annual trends in daily movement distance.

First, linear mixed-effects models were employed to assess the factors influencing home range dynamics by incorporating both fixed and random effects. Among the three candidate models (Table S4), the centered-year model (Model A3) exhibited the best fit (AIC = 4406.9), accounting for 23.1% of the variance through individual random effects. The analysis revealed a significant annual decline in home range size (β = −8.46 km^2^/year, t = −2.75, *p* < 0.01, Figure 3A,C, Table 3). No statistically significant differences were detected across sites, activity period, or age group (all *p* > 0.05). The intercept did not differ significantly from zero (*p* > 0.05).

The optimal mixed-effects model (Model B2, Table 3) revealed significant temporal and activity period-related effects on daily movement distances (Figure 3B). Variation across activity periods was particularly pronounced: individuals exhibited significantly shorter daily movements during the wandering (*p* = 0.003) and wintering periods (*p* = 0.015) compared to the breeding period. Notably, daily movement distances in 2022 were substantially reduced relative to 2015 (β = –1890 ± 772 m, *p* = 0.015), with a similar trend observed in 2019 that approached significance (*p* = 0.052). No other interannual differences were statistically significant. Although some differences in mean movement distances were observed across sites and age groups, these effects were not statistically significant.

Collectively, these findings indicate that the movement ecology of the Crested Ibis is primarily influenced by temporal and behavioral factors, with geographic and demographic variables playing secondary roles.

### 3.3. Habitat Types of Foraging Sites and Related Ecological Factors

#### 3.3.1. Habitat Types of Foraging Sites

**Figure 4:**
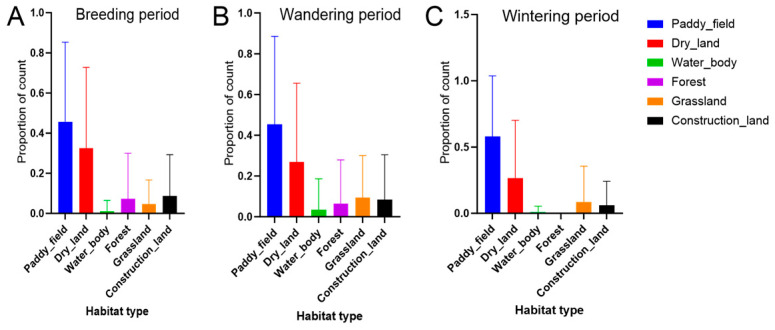
Distribution patterns of foraging sites across various habitat types by record count in (**A**) breeding period; (**B**) wandering period; and (**C**) wintering period.

The habitat classification of foraging sites revealed that paddy fields dominated foraging activity, showing the highest values for both visit frequency and day frequency, followed by drylands (Table 4, Figure 4). During the breeding and wandering periods, construction land and forest areas, often corresponding to nesting regions during the breeding season, accounted for a minor proportion of daytime foraging activity. This pattern was not observed during the wintering period (Figure 4). Across the three activity periods, significant temporal variation in habitat use was detected for several habitat types (Kruskal–Wallis test: *p* < 0.01 for paddy fields; *p* < 0.05 for drylands; Table 4). Pairwise comparisons revealed particularly strong period-based differences in paddy field use (breeding vs. wintering: *p* < 0.01), while drylands also showed significant but less pronounced variation (breeding vs. wintering and wandering vs. wintering: both *p* < 0.05).

The four main habitat types utilized were selected for detailed analysis: paddy fields, drylands, grassland, and forest.

#### 3.3.2. Ecological Factors Influence the Habitat Types of Foraging Sites

**Figure 5:**
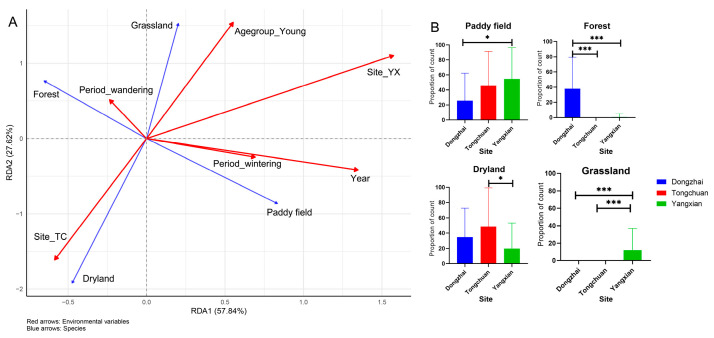
Multivariate analysis of foraging habitat-environment relationships. (**A**) RDA triplot showing environment-habitat associations (red = environmental drivers, blue = habitat types). The arrow length indicates the effect strength. (**B**) Habitat type distribution across three study regions. *** *p* < 0.001, and * *p* < 0.05.

The RDA revealed that spatial location (site) was the dominant factor influencing foraging habitat selection, accounting for the largest proportion of the constrained variance (14.2% total, *p* = 0.001; Figure 5A, Table S5). The YX population was strongly associated with paddy fields, while the TC population was linked to drylands, confirming distinct regional habitat preferences. Additionally, individuals in DZ utilized a higher proportion of woodland areas compared to the other two sites, whereas YX also frequently used grasslands as foraging grounds (Figure 5B).

Age class showed a secondary but notable effect, with young birds preferentially using grasslands, independent of site differences. In contrast, temporal variables (Year and Activity period) had minimal impact, contributing little to habitat choice. These findings highlight that geographic location is the primary driver of habitat use patterns, while age-related differences introduce finer-scale variation.

## 4. Discussion

Analyzing a decade-long GPS tracking dataset enabled the precise identification of nesting, foraging, and roosting sites of Crested Ibis individuals through trajectory reconstruction. Our findings reveal strong site fidelity in nesting, foraging, and roosting behaviors (Table 1, Figure 2). Movement ecology patterns were primarily influenced by temporal and behavioral factors, particularly activity periods (Table 2 and Table 3, and Figure 3). Foraging habitat selection analysis identified spatial heterogeneity as the dominant driver, with cultivated lands, particularly paddy fields and drylands, serving as core foraging zones. In contrast, spatiotemporal variables such as age group had only minor effects (Figure 4 and Figure 5). Clear geographical partitioning was observed, with the TC population preferentially utilizing drylands and the YX population primarily occupying paddy fields.

### 4.1. Conservation Implications in Identifying Nesting, Foraging, and Roosting Sites

Effective species conservation requires a thorough understanding of spatial ecology. While previous research on Crested Ibises has predominantly focused on nesting site dynamics [16], long-term monitoring of wild nests in protected areas of Shaanxi Province (e.g., YX and TC) revealed a gradual shift in nesting site selection toward residential areas between 1981 and 2019 [16]. Our study advances this knowledge by integrating tracking data to quantify both foraging and roosting ranges (Figure 1), providing a comprehensive spatial assessment of the species’ activity range.

Habitat quality plays a critical role in determining reproductive outcomes in the Crested Ibis [17]. Longitudinal data show that pairs breeding in high-quality habitats tend to produce larger broods (three nestlings) with higher offspring quality, evidenced by increased body mass, adult survival, and lifetime reproductive success. In contrast, pairs in degraded habitats predominantly raise smaller broods (two nestlings) with reduced fitness [17]. By integrating GPS tracking data with multi-source environmental datasets, it is identified distinct habitat distribution patterns characterized by preferences for higher elevation areas near woodlands and rivers [13]. In mountainous areas, oak-pine forests demonstrated significant nesting preference within a 100 m radius of breeding cores, particularly occurring in mixed coniferous-broadleaf forest patches along ecotones [18]. Through remote sensing and spatial analysis, it has been quantitatively demonstrated that the Crested Ibis historically exhibited a preference for nest sites clustered in proximity to winter-flooded rice fields, though this habitat dependence has diminished over time [19]. Additionally, nest site selection is most strongly influenced by the combined presence of rice paddies and water bodies [20]. Collectively, these findings clarify the ecological factors shaping habitat selection in the Crested Ibis.

In 2020, the reintroduced population of Crested Ibis in Ningshan County, Shaanxi Province formed a total of 56 breeding pairs. Among these, 30 individuals engaged in breeding dispersal, resulting in 81 documented dispersal events [21]. This behavior facilitated population redistribution toward lower-density habitats. Consequently, conservation efforts must prioritize not only the preservation of core high-quality habitats but also the restoration of marginal areas to support viable brood sizes and function as dispersal sinks. In Ningshan County, Shaanxi Province, nearly 50% of individuals exhibited nest-site fidelity, with data from 2008 to 2018 showing fidelity rates of 45.16% in females and 49.18% in males [22]. In the present study, we further documented high site fidelity in both foraging and roosting locations using comprehensive GPS monitoring data. From a conservation perspective, the consistent use and long-term stability of core breeding, foraging, and roosting areas highlight the importance of prioritizing these high-use zones for protection. Spatial overlay analysis revealed that in YX, many of these critical sites fell outside designated protected areas. This finding suggests that wild populations can thrive in non-protected zones where local communities have developed conservation awareness. These results hold significant implications for Crested Ibis reintroduction programs in other regions of China, such as Deqing (Zhejiang Province) and Yancheng (Jiangsu Province). The species demonstrated adaptability to human-modified environments, combined with established community-based protection measures, offers valuable insights for expanding conservation efforts beyond traditional protected area boundaries.

### 4.2. Implications of Individual Movement Patterns and Foraging Site Selection for Species Conservation

Our analysis of Crested Ibis movement ecology provides critical insights for conservation planning. Previous studies using mark-recapture methods (2000–2002) documented stage-dependent foraging distances (wintering: 2.82 ± 1.49 km; breeding: 0.56 ± 0.68 km; post-breeding: 1.71 ± 0.83 km) [6]. In this study, we present more detailed and temporally extensive movement data. Notably, we identified a significant annual contraction in home range size (β = −8.46 km^2^/year, *p* < 0.01), revealing a previously unreported temporal trend indicative of progressive habitat degradation. This finding aligns with reports of declining farmland habitats in protected areas [23] and the impact of road disturbances, particularly from major highways, on breeding sites [24]. Such range reductions may exacerbate existing threats, as breeding failure has been shown to trigger wider dispersal [25], potentially pushing individuals into suboptimal habitats.

Foraging site selection was primarily driven by spatial heterogeneity, with cultivated lands, particularly paddy fields and drylands, serving as core foraging areas. This pattern is consistent with previous research [20,23,26]. However, we identified a clear regional divergence: TC populations preferentially used drylands, YX populations relied heavily on paddy fields, and DZ individuals primarily foraged in forested areas. This geographic variation reflects known regional differences in habitat selection drivers, ranging from rice field dependence in Ningshan to precipitation-driven patterns in Qianyang [27]. Management strategies should therefore prioritize site-specific habitat conservation, such as maintaining paddy fields in YX and drylands in TC, while also ensuring grassland availability for younger individuals. Regional specialization should also inform reintroduction programs, as demonstrated by variations in human tolerance (e.g., shorter flight initiation distances in plains [28]) and broader trophic niche widths in mountainous regions [29]. Collectively, these findings underscore the need for conservation strategies to integrate both temporal habitat dynamics and spatial heterogeneity in resource use across the species’ range.

## 5. Conclusions

The tracking data provide valuable insights into several key aspects of Crested Ibis ecology, including nesting, foraging, roosting sites, site fidelity, and habitat selection for foraging. However, certain technical limitations should be acknowledged. In mountainous roosting areas, signal loss from tracking devices was common. For example, we experienced an eight-month data gap for individual CAFL011 (January to August 2022), contributing to a data completeness rate of only 81.34% (388 out of 477 individual months with more than 15 days of recording). As such, we cannot rule out the possibility that some individuals without identified nesting sites may have attempted, but failed, to breed during those years. Regarding the detection of breeding behavior, our tracking data successfully identified 15 breeding events out of 32 potential breeding seasons, yielding a detection rate of 46.88%. This rate closely aligns with the average breeding success rate of 54.84% recorded in Ningshan, Shaanxi, from 2008 to 2018 [22], thereby supporting the reliability of our identification methodology. Furthermore, the observed nesting site fidelity reinforces the value of GPS tracking as a robust approach for avian behavioral monitoring. Compared to traditional field surveys, satellite tracking overcomes several operational limitations and significantly enhances conservation efforts for the species.

## Figures and Tables

**Table 1:** Numbers and fidelity of foraging and roosting sites.

		Whole Year	Breeding Period	Wandering Period	Wintering Period
No. foraging sites	N-individual × year	73	46	61	51
	Range	2–149	2–72	1–81	1–29
	Mean	38.08	23.54	24.46	11.45
No. roosting sites	N-individual × year	72	45	60	48
	Range	1–159	1–82	1–68	1–53
	Mean	40	23.96	26.37	13.83
Foraging site fidelity	Fidelity range (%)	5.13–84.21	7.27–94.87	7.08–100	9.14–100
	Fidelity mean (%)	25.27	29.89	28.09	40.54
Roosting site fidelity	Fidelity range (%)	6.57–100	10.53–100	7.36–100	10.95–100
	Fidelity mean (%)	26.11	31.91	30.55	42.70

**Table 2:** Home range size and average daily distance moved by individual Crested Ibises.

Types	Period	N (Individual × Month)	Median	SD	Range
Area_95 (km^2^)	Breeding	197	7.76	195.57	0.00–2004.23
	Wandering	184	15.45	170.01	0.01–1324.99
	Wintering	108	9.18	107.94	0.01–640.22
	Whole year	489	10.87	169.89	0.00–2004.23
Average daily distance (m)	Breeding	202	2952.25	5762.40	29.85–57,366.71
	Wandering	214	3081.73	3113.71	27.83–17,546.70
	Wintering	116	3023.81	3383.15	146.49–21,470.40
	Whole year	532	3043.80	4361.32	27.83–57,366.71

**Table 3:** Linear mixed model fixed effects of movement pattern dynamics. Model parameters of lmer models in Table S3. * *p* < 0.05, ** *p* < 0.01.

Model	Factor	β (SE)	t Value	*p* Value
Model A3: lmer(area_95 ~ Site + Year_centered + Age group + Activity period + (1|ID)	Year_centered	−8.46 (3.08)	−2.750	**
Model B2: lmer(avg_daily_distance ~ Site + Year + Age group + Activity period + (Season|ID)	Year_2019	−971.87 (497.56)	−1.953	0.052
	Year_2022	−1890.15 (771.83)	−2.449	*
	Activity period wandering	−601.89 (203.89)	−2.952	**
	Activity period wintering	−584.07 (238.58)	−2.448	*

**Table 4:** Statistical analysis of visit proportion by foraging site habitat type. * *p* < 0.05, ** *p* < 0.01, NS no significance.

Habitat Type		Paddy Land	Dry Land	Water Body	Grass Land	Construction Land	Forest
**Mean ± SD**	Breeding	0.457 ± 0.397	0.325 ± 0.402	0.011 ± 0.053	0.046 ± 0.121	0.087 ± 0.205	0.073 ± 0.227
	Wandering	0.453 ± 0.432	0.268 ± 0.387	0.035 ± 0.151	0.094 ± 0.206	0.084 ± 0.220	0.065 ± 0.215
	Wintering	0.579 ± 0.459	0.266 ± 0.435	0.008 ± 0.047	0.086 ± 0.269	0.061 ± 0.181	0 ± 0
Kruskal–Wallis test		**	*	NS	NS	NS	NS
Wilcoxon paired test ^a^	Breeding vs. Wandering	NS	NS	-	-	-	-
	Breeding vs. Wintering	**	*	-	-	-	-
	Wandering vs. Wintering	NS	*	-	-	-	-

Note: ^a^, when the initial Kruskal–Wallis test showed no significant differences (*p* ≥ 0.05), no further pairwise testing was conducted, as this hierarchical approach ensures statistical robustness while minimizing false discoveries in the ecological dataset.

## Data Availability

All survey data during this study are included in this published article and its Supplementary Material.

## Supplementary Materials

The following supporting information can be downloaded at https://www.mdpi.com/article/10.3390/ani15172555/s1: Table S1: Individuals used in this study. Monitoring time of breeding period longer than 3 month were considered as one; Table S2: No. of roosting and foraging sites identified per year-individual. Table S3: Nest Site identification and post-validation; Table S4: Model parameters of lmer models; Table S5: Multivariate analysis of foraging habitat associations: RDA Results for ecological communities.
